# Design of a Microwave Power Detection System in the 5G-Communication Frequency Band

**DOI:** 10.3390/s21082674

**Published:** 2021-04-10

**Authors:** Qingying Ren, Wen Zuo, Jie Xu, Leisheng Jin, Wei Li, Debo Wang

**Affiliations:** College of Electronic and Optical Engineering & College of Microelectronics, Nanjing University of Posts and Telecommunications, Nanjing 210023, China; rqy@njupt.edu.cn (Q.R.); b17021132@njupt.edu.cn (W.Z.); jiexu@njupt.edu.cn (J.X.); jinls@njupt.edu.cn (L.J.); liw@njupt.edu.cn (W.L.)

**Keywords:** 5G communication, microwave power, detection system, dynamic range, sensitivity

## Abstract

At present, the proposed microwave power detection systems cannot provide a high dynamic detection range and measurement sensitivity at the same time. Additionally, the frequency band of these detection systems cannot cover the 5G-communication frequency band. In this work, a novel microwave power detection system is proposed to measure the power of the 5G-communication frequency band. The detection system is composed of a signal receiving module, a power detection module and a data processing module. Experiments show that the detection frequency band of this system ranges from 1.4 GHz to 5.3 GHz, the dynamic measurement range is 70 dB, the minimum detection power is −68 dBm, and the sensitivity is 22.3 mV/dBm. Compared with other detection systems, the performance of this detection system in the 5G-communication frequency band is significantly improved. Therefore, this microwave power detection system has certain reference significance and application value in the microwave signal detection of 5G communication systems.

## 1. Introduction

Microwave power detection plays an important role in level detection, power monitor and gain control [1,2,3]. Generally, there are three main kinds: thermoelectric microwave power sensors, capacitive microwave power sensors and coupling-type microwave power sensors.

The thermoelectric microwave power sensor was firstly proposed by DeHe et al. [4,5], which acted as a receiving terminal and detects the microwave power based on Seebeck effect. In 2012, Liu et al. established a thermal equivalent model of the sensor and optimized its sensitivity [6]. In 2013, Yi et al. proposed a 2-D mathematical analytical model of the sensor and optimized the sensor’s sensitivity to 0.26 mV/mW [7]. In 2014 and 2015, the structure of thermoelectric microwave power sensor was optimized by Zhang et al. [8,9], and its sensitivity could reach 0.671 mV/mW. However, the dynamic measurement range of the thermoelectric microwave power sensor is not ideal, it was only about 20 dB.

The capacitive microwave power sensor, firstly proposed by Fernandez et al. [10,11], was based on sensing the electrostatic force generated between the transmission line and the cantilever beam. Compared with the thermoelectric microwave power sensor, capacitive microwave power sensor has the advantage of high overload power and on-line detection. Meanwhile, the microwave performance of the power sensor is good in X-band. In 2014, Han et al. established an analytical model of a fixed beam and the S parameters of the power sensor was analyzed and optimized [12]. In 2018 and 2019, the structure of the capacitive microwave power sensor was optimized by Yan [13] and Chu [14]. The overload power of the sensor was improved up to 1 W, but the dynamic measurement range was only 20 dB and the minimum detection power was 100 mW, which was poor.

The coupling-type microwave power sensor was proposed firstly by Han et al. [15,16]. The clamped beam acted as a coupler and the microwave signal was detected by a thermoelectric power sensor. Compared with the above two types MEMS power sensors, the dynamic detection range of the coupling-type power sensor is much larger. In 2015, Zhang et al. optimized the S-parameters of the coupling-type power sensor [17], but the sensitivity of the proposed senor was only 29.5 μV/mW. In the work of Yi et al. [18,19], the dynamic measurement range of coupling-type microwave power sensor was optimized up to 32 dB. However, its sensitivity was just 0.124 mV/mW in the X-band.

It is recognized that the highest dynamic range of the existing detection systems is only about 30 dB, which is not ideal for the detection module of 5G-communication system. Furthermore, the measurement sensitivity and the minimum detection power of these power sensors also need to be improved. In our previous work, a microwave power detection system based on micro-electromechanical system (MEMS) thermoelectric power sensor was proposed [20], and a pivoted mechanical model of capacitive MEMS microwave power sensor was established to optimize the relative position of the measuring electrode. In addition, an LTE-band microwave power detection system based on a Schottky diode was proposed in the 4G communication frequency band [21] and the dynamic range and the minimum detection power of system was improved.

In this work, a novel power detection system is designed in the 5G communication frequency band. The microwave power detection system integrates a receiving antenna, power detector and MCU. Compared with the previous detection systems, the sensitivity, the minimum detection power and the dynamic measurement range of this detection system are all significantly improved. In Section 2, three main modules of this detection system are designed and researched. In Section 3, the return loss of signal receiving module is measured to study the matching characteristics of this detection system. The measurement error and the dynamic range are measured to study the performance of this detection system. Finally, some conclusions are drawn and the application prospects of this detection system are put forward in Section 4.

## 2. Principle and Theory

**Figure 1:**
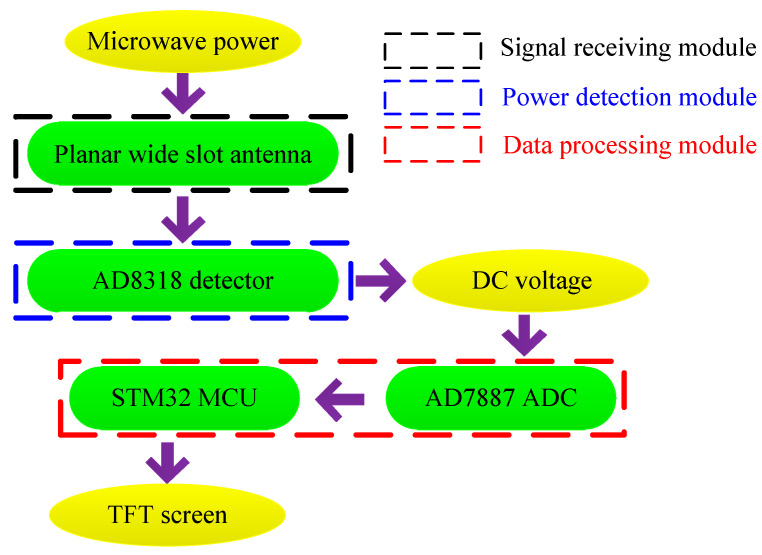
Principle of this detection system.

This microwave power detection system is composed of three modules: signal receiving module, power detection module and data processing module. The principle of this detection system is shown in Figure 1. Firstly, the microwave power signal is received by the planar slot antenna and transmitted to the power detection module. Then, the detection chip AD8318 converts the input microwave power into a DC voltage, and the output voltage is transmitted to the data processing module. After that, the AD7887 A/D chip converts the analog voltage into a digital signal and transmits it to the STM32 microcontroller. Finally, the output voltage of the detector will be obtained, and the microwave power will be displayed with the TFT capacitive screen.

### 2.1. The Signal Receiving Module

**Figure 2:**
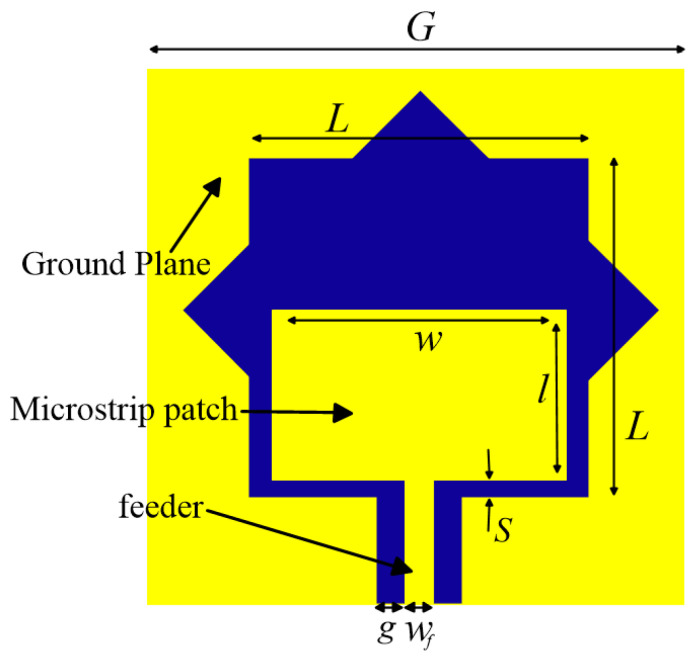
Structure of planar wide slot antenna.

In this module, a planar wide slot antenna fed by coplanar waveguide is designed to receive and transmit microwave power signals, which is mainly composed of a substrate, a ground plane, a microstrip patch and a feeder as shown in Figure 2. The frequency band of this antenna is from 1.8 GHz to 5 GHz.

When the thickness and the dielectric constant of the substrate are determined, the width *w* and the length *l* of the microstrip patch can be obtained according to Equations (1) and (2) [22]:

(1) $$w=\frac{c}{2f}{\left(\frac{\varepsilon_{r}+1}{2}\right)}^{-1/2}$$

(2) $$l=\frac{0.5\lambda_{0}}{\sqrt{\varepsilon_{e}}}-0.824h\frac{\left(\varepsilon_{e}+0.3)(w+0.264h\right)}{\left(\varepsilon_{e}-0.258)(w+0.813h\right)}$$

where *c* is the speed of light, *f* is the resonant frequency of the antenna, *ε_r_* is the dielectric constant of the substrate, *λ_0_* is the wavelength in vacuum, *ε_e_* is the effective dielectric constant, and *h* is the thickness of the substrate.

The effective dielectric constant *ε_e_* and the width of ground *G* can be obtained according to Equations (3) and (4) [23]:

(3) $$\varepsilon_{e}=\frac{\varepsilon_{r}+1}{2}+\frac{\varepsilon_{r}-1}{2}{\left(1+\frac{12h}{w}\right)}^{-1/2}$$

(4) $$G=L+0.2\lambda_{g}$$

where *L* is the gap size, and *λ_g_* is the wavelength in the medium.

*λ_g_* can be obtained as [24]:

(5) $$\lambda_{g}=\frac{\lambda_{0}}{\sqrt{\varepsilon_{r}}}$$

In order to expand the antenna’s frequency band and improve the matching performance, the other three sides of the planar wide slot antenna are designed with the Koch fractal technology except the input side [25]. Therefore, there are some isosceles right triangles in the slot. The HFSS software is used to optimize the relevant size of the antenna. The return loss of the antenna is lower than −10 dB in the band of 1.8 GHz to 5 GHz. The structure size of this antenna is shown in Table 1.

### 2.2. The Power Detection Module

**Figure 3:**
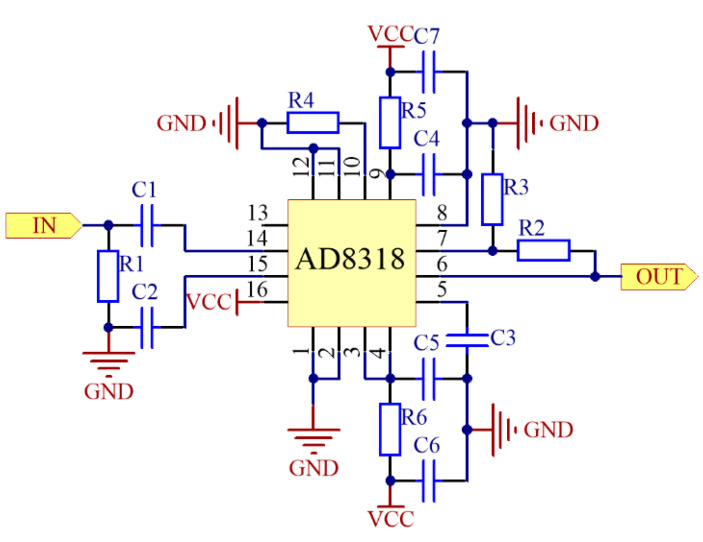
Schematic diagram of power detection module.

In this system, the power detection module is mainly composed of the ADI (Norwood, MA, USA) logarithmic detector chip AD8318 and its circuits, as shown in Figure 3. The detection frequency band of AD8318 covers the 5G communication band. Therefore, the AD8318 is chosen as the detection chip of this system. At the input port of power detection module, the microwave power signal will firstly go through the impedance matching network composed of R1, *C*1 and *C2*. In order to realize the broadband impedance matching, the value of R1 is 52.3 Ω. *C*1 and *C*2 are both 1 nF.

The microwave power is detected by this power detection module, and a DC voltage signal is output from the pin 6. The output DC voltage can be expressed as [26]:

(6) $$V_{dc}=X\cdot 20\cdot \log_{10}(\frac{V_{in}}{2.239})=2.1+X\cdot P_{in}$$

where *X* is a variable coefficient, which can be controlled by changing the values of R2 and R3. *V_in_* is the effective value of the input signal and *P_in_* is the input power.

The grounding capacitor *C*3 connected to the pin 5 forms a low-pass filter with the internal circuit of the chip, which can filter the high-frequency noise of the output DC signal. The value of *C*3 can be obtained according to Equation (7) [22]:

(7) $$C3=\frac{1}{\pi \times 3.13k\Omega \times BW}-1.5pF$$

where *BW* is the cut-off frequency of the low-pass filter and the value of *C*3 in this design is 1 μF.

As for other components in the power detection module, *C*4, *C*7 and *C*5, *C*6 are two pairs of by-pass capacitors connected with the AD8318’s power pin. Their values are both 100 pF//100 nF. R5 and R6 are 0 Ω magnetic beads. The role of resistors can filter the ripple of the power supply and make the chip work more stably. The range of R2 and R3 is 0~10 kΩ, and the ratio of R2 and R3 determines the measurement range of the power detection module.

### 2.3. The Data Processing Module

**Figure 4:**
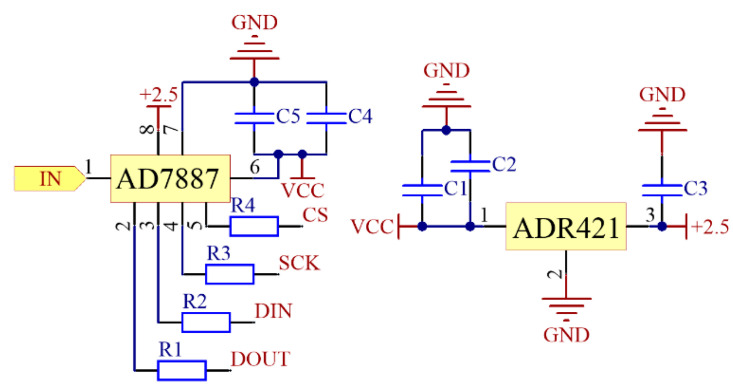
Schematic diagram of AD7887 and ADR421.

In order to achieve higher accuracy and more stable temperature drift characteristics of the data processing module, the ADI 12-bit micro-power chip AD7887 is used to realize the data processing as shown in Figure 4. The reference voltage of AD7887 is provided by an ultraprecision and low noise voltage reference chip ADR421. The data processing and analysis is realized by the microcontroller STM32. The function of the TFT screen is to record the experiment data. In the measurement process, the TFT screen will display the input power and the output voltage.

The DC voltage is transmitted into AD7887 from pin 1 firstly and then converted into a digital signal. Then the chip transmits the digital signal into the MCU by R1~R4. R1~R4 are 100 Ω resistors, which can reduce the digital noise and make the sampling results more stable. The 2.5 V reference voltage generated from ADR421 is connected to AD7887 with a grounding capacitor *C*3. The value of *C*3 is 100 nF and it can reduce the ripple of the reference voltage. *C*1, *C*2 and *C*4, *C*5 are two pairs of by-pass capacitor connected with the VCC of ADR421 and AD7887, and their values are both 100 nF//10 μF.

Assuming that the 12 Bit binary code output by A/D chip is *d*_11_~*d*_0_ from high to low, and *V_out_* is the output of decimal digital voltage. Then there is a relationship of *V_out_* with *V_dc_* as:

(8) $$V_{out}=\sum_{i=0}^{11}(d_{i}\cdot \frac{2^{i}\cdot V_{ref}}{2^{12}})+V_{offset}=A_{adc}\cdot \frac{V_{dc}}{V_{ref}}\cdot 2^{12}+V_{offset}$$

where *V_ref_* is the reference voltage of A/D chip with a 2.5 V, *A_adc_* is the pre-amplifier gain of SAR_ADC comparator and *V_offset_* is the offset voltage of the comparator.

So, the relationship of *V_out_* with *P_in_* can be obtained as:

(9) $$V_{out}=A_{adc}\cdot \frac{2^{12}}{V_{ref}}\cdot \left(2.1+X\cdot P_{in}\right)+V_{offset}=V_{intercept}+S\cdot P_{in}$$

It can be seen that there is a linear relationship of the output voltage *V_out_* with the input power *P_in_*. *V_intercept_* is the intercept of the line and *S* is the slope of the line, namely the sensitivity of this detection system.

**Figure 5:**
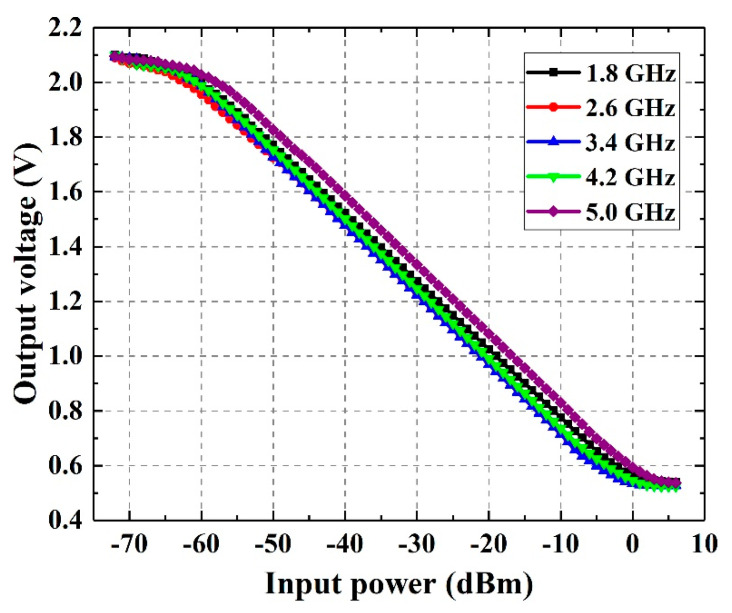
Relationship of the input power with the output voltage.

In this work, the linearity correction algorithm is used for MCU to improve the measurement accuracy and the dynamic range of system. In the power detection module, the input power is converted into output voltage and the measured I-O performance is shown in Figure 5.

When the input power is too small or too large, the linearity of the output voltage will deteriorate. In order to correct the large deviation part and extend the dynamic range, the I-O model is divided into three parts as shown in Equation (10):

(10) $$P_{in}=\left\{ \begin{array}{l} a_{1}+b_{1}\cdot V_{out}+c_{1}\cdot V_{out}^{2}+d_{1}\cdot V_{out}^{3}\;,\;V_{out}>1.95V\\ a_{2}+b_{2}\cdot V_{out}+c_{2}\cdot V_{out}^{2}\;,\;0.65V\leq V_{out}\leq 1.95V\\ a_{3}+b_{3}\cdot V_{out}+c_{3}\cdot V_{out}^{2}+d_{3}\cdot V_{out}^{3}\;,\;V_{out}<0.65V \end{array}\right.$$

where *a*_i_, *b*_i_, *c*_i_, *d*_i_ are the coefficients and their values are shown in Table 2. The related coefficients are calculated by polynomial fitting the data in Figure 5.

Since the value of *V_offset_* is unknown in Equations (8) and (9), *V_offset_* is ignored in order to obtain a concise expression. However, the output of the microwave power detection system is calibrated with the experiment results, so *V_offset_* has been considered and corrected. Thus the measurement error caused by *V_offset_* has been eliminated. Finally, the more accurate power detection and higher dynamic range of this detection system are realized. The schematics of the MCU board and relevant code are provided in the Supplementary File.

## 3. Measurements and Discussions

**Figure 6:**
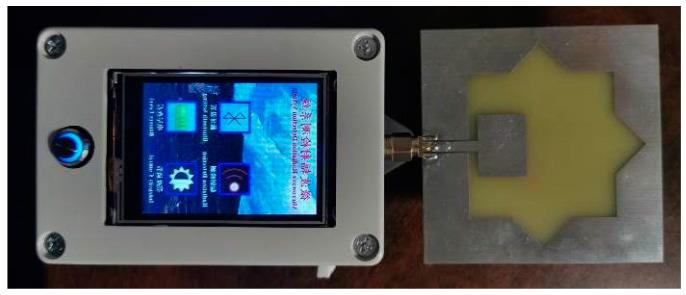
Photo of the microwave power detection system.

The layout design and the PCB design of this power detection system are realized with Altium Designer 16.0 (Altium, Sydney, Australia). According to the design, a Grounded Coplanar Waveguide (GCPW) is chosen as the matching transmission line between the signal receiving module and the power detection module to reduce the return loss. The PCB design is made of FR-4 fiberglass board, and the thickness of the substrate and copper foil is 1.6 mm. The Advanced Design System (ADS) software (KEYSIGHT, Santa Rosa, CA, USA) is used to design the sizes of GCPW, and the characteristic impedance of the transmission line is 50 Ω. The microwave power detection system is designed as shown in Figure 6.

**Figure 7:**
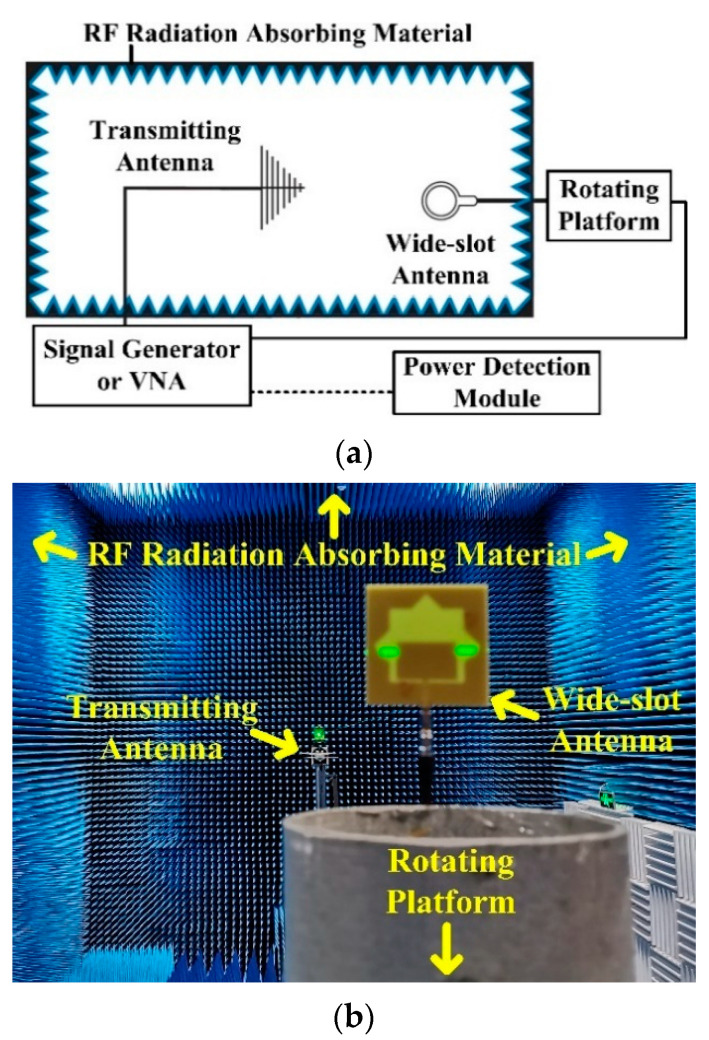
Measurement Environment. (**a**) Connection frame; (**b**) Photo of the environment.

As shown in Figure 7, the measurement of the power detection system is finished in the microwave anechoic chamber. The vector network analyzer (VNA) is connected with the wide-slot antenna when measuring the S11 parameter. When measuring the directivity and the gain of antenna, the transmitting antenna and the wide-slot antenna are connected to the VNA and the rotating platform, respectively. When measuring the error and the dynamic range of system, the signal generator is linked to the power detection module and the detected input power is shown in the TFT screen.

### 3.1. Measurement of S11 Parameter

**Figure 8:**
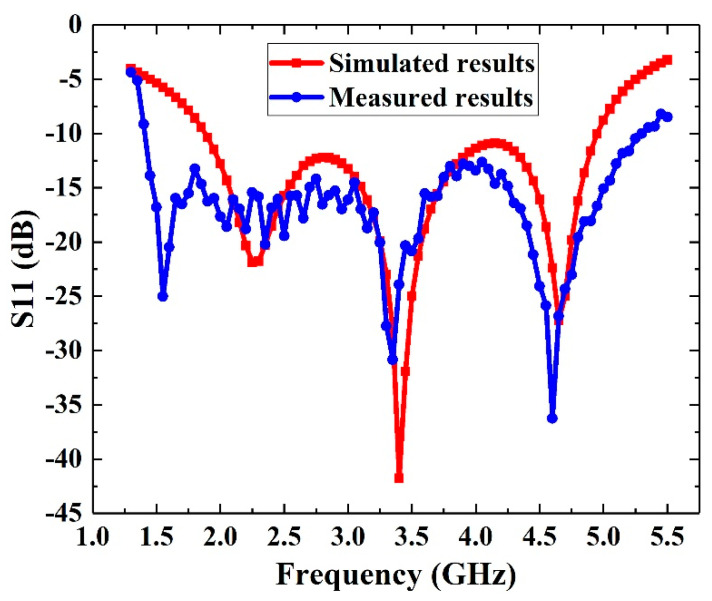
Comparison between simulated and measured results.

A E5071C vector network analyzer (KEYSIGHT, Santa Rosa, CA, USA) is used to measure the return loss (S11 parameter) of the signal receiving module. In the frequency band range from 1.3 GHz to 5.5 GHz, the simulated results and the measured results are compared as shown in Figure 8. It can be seen that the measured results of S11 are basically consistent with the simulated results. There are only some minor deviations between the simulated results and the measured results. The reason is that the dielectric constant of FR-4 board has some errors on the surface and in the body. At the same time, the size of the antenna will also produce some minor errors in the fabrication. However, the signal receiving module can meet the requirements of covering 5G communication frequency band (1.8~5 GHz). The measured results of the signal receiving module is less than −10 dB in the range of 1.4 GHz to 5.3 GHz.

### 3.2. Measurement of Directivity and Gain

**Figure 9:**
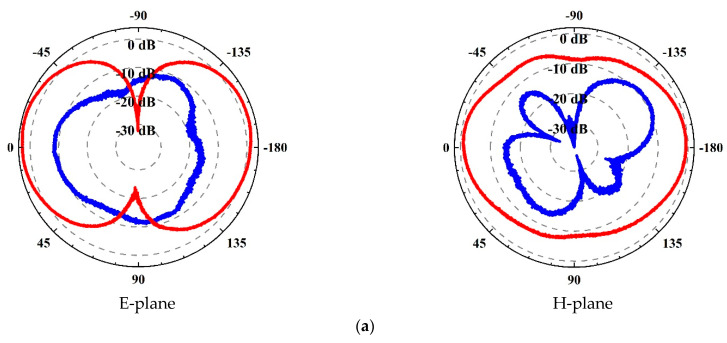

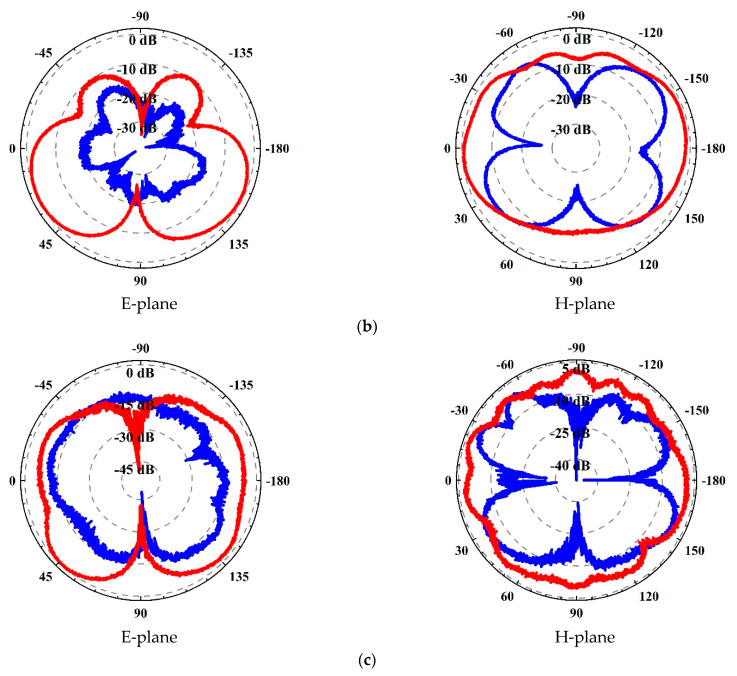
Normalized E-plane and H-plane radiation patterns. (**a**) *f =* 2 GHz; (**b**) *f =* 3.5 GHz; (**c**) *f =* 5 GHz. Red color indicates coplanar polarization, and blue color indicates cross polarization.

The antenna is fixed in the rotating platform when measuring the directivity and gain. After calibration, the platform rotates slowly for 360 degrees and the measured results are recorded at each angle. The measured normalized E-plane and H-plane radiation patterns of the antenna are shown in Figure 9. When the frequency is at 2 GHz, the direction of the maximum gain is 0° and 180°. As the frequency increases, the direction of the maximum gain gradually changes, which is caused by different matching characteristics. Meanwhile, the cross-polarized radiation of the antenna is at least 10 dB weaker than the co-planar polarized radiation in the direction of the E-plane. The cross-polarized radiation in the H-plane is relatively larger than that in the E-plane. To sum up, the main lobe Angle of antenna pattern is large and the directionality of the antenna is not obvious, which is conducive to the antenna to receive microwave radiation signals better. Therefore, the directional parameters of the designed antenna can meet the requirements of the detection system.

**Figure 10:**
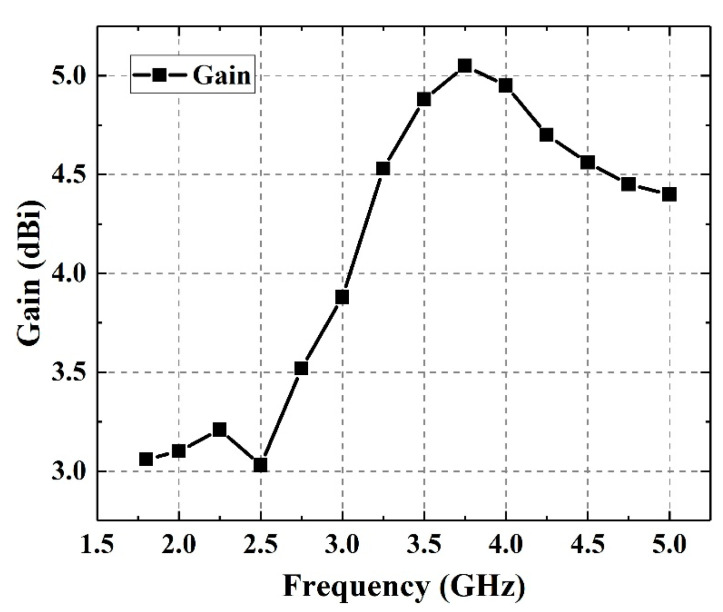
Measured results of antenna gain with frequency.

The measured gain of the antenna is shown in Figure 10. It can be seen that the antenna gain has a range of 3.03 to 5.05 dBi in the frequency band and the fluctuation is less than 3 dB. The gain of the antenna at 2, 3.5 and 5 GHz is 3.1, 4.88 and 4.4 dBi, respectively. In general, the directivity of the designed antenna is not obvious, so the gain of the antenna is not large. However, the gain fluctuation of the antenna is small in the frequency band range, so the performance of the detection system will not be overly sensitive to the frequency and the gain parameters of the antenna can basically meet the requirements of the detection system.

### 3.3. Measurement of Error and Dynamic Range

A MG3710A Vector Signal Generator (Anritsu, San Francisco, CA, USA) is used to measure the error and the dynamic range of this detection system. The measurement error of the detection system can be calculated as:

(11) $$E=\frac{\left|P_{mea}-P_{out}\right|}{P_{out}}\times 100\%$$

where *E* is the measurement error of the system, *P_out_* is output power from the signal generator and *P_mea_* is the detected power.

**Figure 11:**
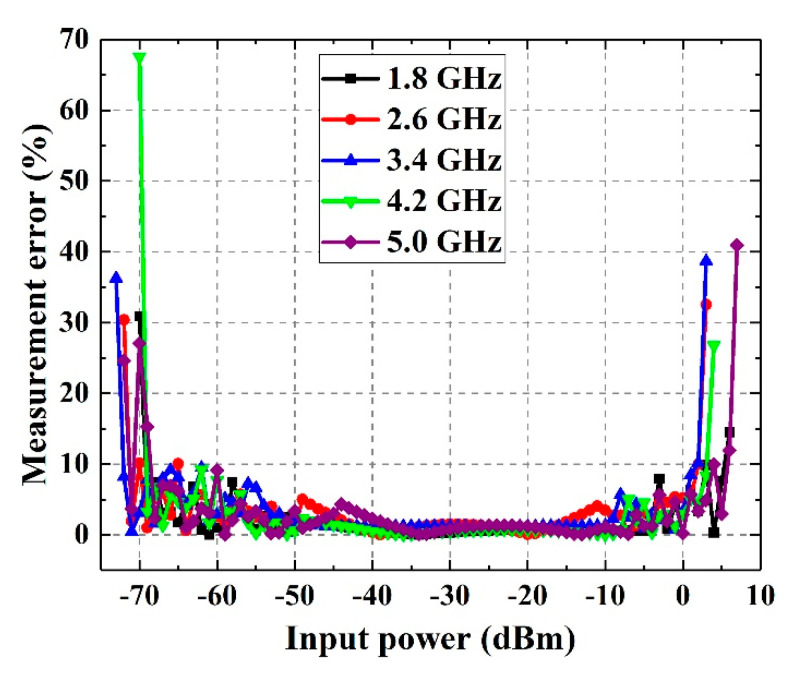
Measurement error of the detection system.

In the frequency band ranges from 1.8 GHz to 5 GHz, when the input microwave signal increases from −75 dBm to 10 dBm, the measurement error of the power detection system is shown in Figure 11. It can be found that when the input power is too small or too large, the measurement error of the system will grow rapidly. The reason is that when the input power approaches the detection limit of AD8318, the output voltage of AD8318 will produce a relatively large deviation from linearity, and the error of AD8318 itself will also gradually increase.

**Figure 12:**
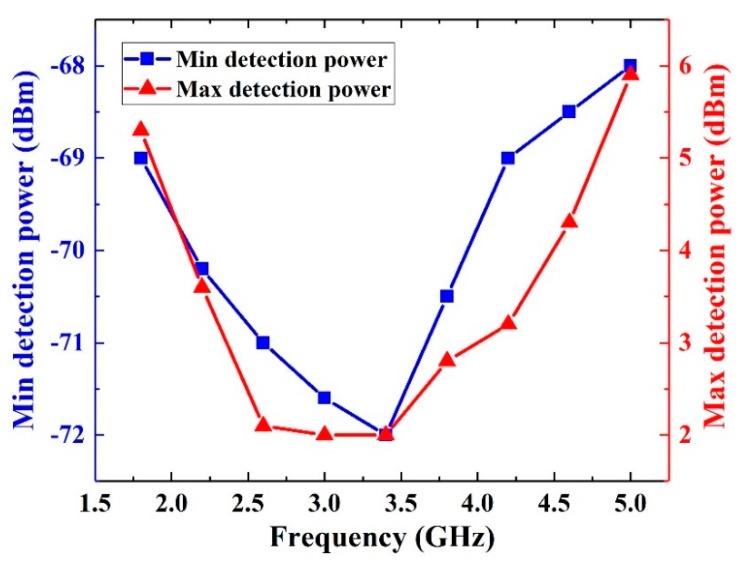
Dynamic range of the detection system.

In order to evaluate the performance of the proposed microwave power detection system, we assume that when the measurement error is lower than 10%, the input power is within the measurement range. Therefore, based on the relevant data in Figure 11, the maximum and the minimum detection power of this detection system are shown in Figure 12. It can be seen that the maximum and the minimum detection power both drop firstly and then grow with the frequency. The reason is that the parasitic effect of GCPW transmission line becomes more obvious with the frequency, which leads to the deterioration of GCPW’s matching characteristics. As a result, the microwave power transmitted into the detection system decreases. Meanwhile, the detection limit of AD8318 has a downward trend with the frequency. The characteristics of AD8318 plays the main role when the frequency is low, but with the increasing of the frequency, the parasitic effect of GCPW plays the main role. Therefore, the minimum and the maximum measured power of the detection system drops firstly and then grows with the frequency.

To sum up, the dynamic range of this power detection system is −68 dBm to 2 dBm. The measured sensitivity is 22.3 mV/dBm (22.3 mV/mW), and the measurement error is less than 10% in 5G communication frequency band. Table 3 compares the dynamic detection range, minimum detection power and sensitivity between this detection system and other detection systems. Compared with other detection systems, this microwave power detection system has significant improvement in dynamic measurement range, minimum detection power and sensitivity in 5G-communication frequency band. On the other hand, the detection system also has its limitations. Firstly, compared with other microwave sensors, the detection frequency band of the system is limited to 5 GHz and cannot be extended to higher frequency. Meanwhile, the area and the power consumption of this system is larger than the MEMS microwave power sensors.

## 4. Conclusions

In this work, a microwave power detection system based on a AD8318 chip is proposed for the 5G communication frequency band. Compared with other microwave power sensors, this detection system can improve the dynamic range, the detection sensitivity and the minimum detection power. Based on the Koch fractal technology, the planar wide-slot antenna proposed in the work covers the entire 5G communication frequency band. Meanwhile, it has a good performance in the in-band gain fluctuation and the directivity. Experiment reveals that the dynamic measurement range of the system is 70 dB, the minimum detection power is −68 dBm and the output sensitivity is 22.3 mV/dBm. Based on the experiment results, the linear correction algorithm is written into the MCU and the measurement error of this system is less than 10% in the 5G communication frequency band. Thus, this microwave power detection system can achieve high accuracy of power detection. Therefore, this detection system has a potential for microwave detection applications with high dynamic range and minimum detection power in 5G communication systems.

## Figures and Tables

**Table 1:** Structure size of this antenna.

**Feature**	**Symbol**	**Value**
Width of the ground	*G*	72 mm
Length of slot	*L*	44 mm
Distance between patch and ground	*S*	2.9 mm
Width of microstrip patch	*w*	19.02 mm
Length of microstrip patch	*l*	14.88 mm
Distance between feeder and ground	*g*	0.3 mm
Width of feeder	*w_f_*	2.18 mm

**Table 2:** Values of the coefficients.

**Frequency**	**1.8 GHz**	**2.6 GHz**	**3.4 GHz**	**4.2 GHz**	**5 GHz**
*a*1	22,070.09	16,075.62	239,434.56	181,312.14	98,472.62
*b*1	−33,803.43	−24,962.33	−352,678.4	−271,412.7	−147,548.6
*c*1	17,228.35	12,892.29	173,143.09	135,401.76	73,677.41
*d*1	−2929.58	−2222.78	−28,338.75	−22,519.33	−12,267.88
*a*2	21.124	17.403	18.087	18.581	21.747
*b*2	−39.911	−37.091	−39.33	−38.594	−37.55
*c*2	−0.184	−1.134	−0.016	−0.293	−0.911
*a*3	14,284.09	2352.94	5254.66	1332.58	2944.75
*b*3	−74,069.41	−11,750.83	−27,328.92	−6520.78	−14,659.35
*c*3	128,105.33	19,594.62	47,427.82	10,697.02	24,387.03
*d*3	−73,903.14	−10,930.86	−27,483.61	−5871.38	−13,558.75

**Table 3:** Comparison of dynamic range, minimum detection power and sensitivity.

**Reference**	**Dynamic Range**	**Minimum Detection Power**	**Sensitivity**
[6]	20 dB	0 dBm	0.671 mV/mW
[10]	26 dB	20 dBm	4.8 aF/mW
[11]	20 dB	20 dBm	2.8 aF/mW
[14]	32 dB	−10 dBm	0.124 mV/mW
This work	70 dB	−68 dBm	22.3 mV/mW

## Data Availability

We choose to exclude this statement.

## Supplementary Materials

The following are available online at https://www.mdpi.com/article/10.3390/s21082674/s1, Supplementary File: The schematics of the MCU board and relevant code.
