# Supplementary material for: Design of a Microwave Power Detection System in the 5G-Communication Frequency Band
Source: Sensors (Basel). 2021 Apr 10;21(8):2674. doi: 10.3390/s21082674 (PMC8068901; doi:10.3390/s21082674)
Supplement: Supplementary file 1 [file sensors-21-02674-s001.pdf]

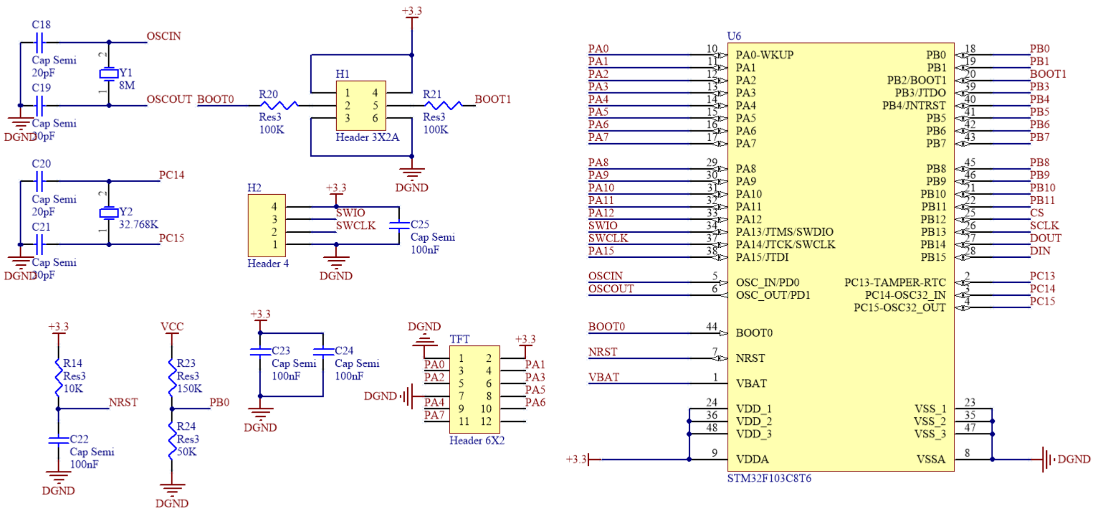

Figure.1 Schematic of MCU

This code is the master control code of the STM32F103C8T6,which is the MCU in the proposed microwave power detection system in 5G-communication frequency band. The linear correction algorithm, driver of the ADC chip AD7747 and control of the TFT LCD touch screen are realized in the MCU by the following codes. Meanwhile, these codes also realize the wireless data transmission by Bluetooth, battery power display and usage time prediction by another ADC channel.

```
#include "led.h"
#include "delay.h"
#include "sys.h"
#include "usart.h"
#include "lcd.h"
#include "adc.h"
#include "usart3.h"

extern u32 receive_data;
u8 key,beep;
float Detected_power;
float Battery_rate_f;
int Battery_rate;
float percentage=100;
u16 button;
int BLE;
u16 adcx_2;
float temp_2;
u16 adcx;
float temp;
int BAT_Usagetime[]={0,5,10,16,21,26,30,36,43,50,57,64,71,80,85,91,97,105,113,120,126,
```

```
132,140,145,152,157,163,170,175,180,186,190,197,200,204,208,214,219,221,226,230,

233,237,239,242,247,250,252,255,259,263,266,269,272,275,279,281,284,287,289,291,

294,296,298,299,301,303,305,307,310,312,314,318,323,326,329,332,335,337,339,342,

344,347,350,352,357,359,363,366,368,371,373,376,378,380,383,386,389,392,394,402};

void HMISends(char *buf1);
void HMISendb(u8 buf);
u32 HMI_Pow(u8 n);
void HMI_SendNum(USART_TypeDef* USARTx,u8 *data,u8 len,u16 Num,u8 len2);
void beepms(u16 va);
void HMISendstart(void);
void Powerdetection(void);
void Bluebooth(void);
void Batterydisplay(void);

int main(void)
{
	SystemInit();
	delay_init();
		NVIC_PriorityGroupConfig(NVIC_PriorityGroup_2);
	uart_init(9600);
	uart3_init(9600);
	HMISendstart();
	LED_Init();
	Adc1_Init();
	Adc2_Init();
		LED1=1;
	BLE=0;
	button=0;
		receive_data=0;
		USART3_RX_STA=0;
	delay_ms(100);
	adcx=Get_Adc1_Average(ADC_Channel_1,100);
	temp=(float)adcx*(3.3/4096);
	Detected_power=(24.36-56.75*(temp)+14.22*(temp*temp)-4.07*(temp*temp*temp))*(-10);
		HMI_SendNum(USART3,"button0.x0.val=-",16,(int)Detected_power,3);
	adcx_2=Get_Adc2_Average(ADC_Channel_9,120);
	temp_2=(float)adcx_2*(3.3/4096);
	Battery_rate_f=((1-(8.15-temp_2*2.55)/1.03)*100);
	Battery_rate=(int)Battery_rate_f;
	if(Battery_rate>100)
```

```
        Battery_rate=100;
        if(Battery_rate<=0)
            Battery_rate=0;
    percentage=Battery_rate;
    HMI_SendNum(USART3,"button3.n0.val=",15,Battery_rate,3);
    HMI_SendNum(USART3,"button3.n1.val=",15,BAT_Usagetime[Battery_rate]/60,2);
    HMI_SendNum(USART3,"button3.n2.val=",15,BAT_Usagetime[Battery_rate]%60,2);
    while(1)
    {
        if(receive_data!=0)
         {
           button=receive_data;
                   Bluebooth();
           receive_data=0;
           USART3_RX_STA=0;
         }
        Powerdetection();
        Batterydisplay();
        delay_ms(200);
    }
}

void HMISends(char *buf1)
{
    u8 i=0;
    while(1)
    {
     if(buf1[i]!=0)
        {
            USART_SendData(USART3,buf1[i]);
            while(USART_GetFlagStatus(USART3,USART_FLAG_TXE)==RESET){};
            i++;
        }
     else
     return ;

        }
    }

u32 HMI_Pow(u8 n)
{
    u32 result=1;
    while(n--)result*=10;
    return result;
```

```
}

void HMI_SendNum(USART_TypeDef* USARTx,u8 *data,u8 len,u16 Num,u8 len2)
{
        u8 t;
 for(t=0;t<len;t++)
        {
            while(USART_GetFlagStatus(USARTx,USART_FLAG_TC)!=SET);
            USART_SendData(USARTx,data[t]);
        }
for(t=0;t<len2;t++)
{
           while(USART_GetFlagStatus(USARTx,USART_FLAG_TC)!=SET);
           USART_SendData(USARTx,'0'+(Num/HMI_Pow(len2-t-1))%10);



}

            while(USART_GetFlagStatus(USARTx,USART_FLAG_TC)!=SET);
            USART_SendData(USARTx,0XFF);


            while(USART_GetFlagStatus(USARTx,USART_FLAG_TC)!=SET);
            USART_SendData(USARTx,0XFF);


            while(USART_GetFlagStatus(USARTx,USART_FLAG_TC)!=SET);
            USART_SendData(USARTx,0XFF);

}

void HMISendb(u8 k)
{
    u8 i;
     for(i=0;i<3;i++)
     {
     if(k!=0)
        {
            USART_SendData(USART3,k);
            while(USART_GetFlagStatus(USART3,USART_FLAG_TXE)==RESET){};
        }
     else
     return ;
```

```
    }
}

void beepms(u16 va)
{
    beep=1;
    delay_ms(va);
    beep=0;
}

void HMISendstart(void)
{
     delay_ms(200);
     HMISendb(0xff);
     delay_ms(200);
}
void Powerdetection(void)
{
    if(button==1)
    {
        adcx=Get_Adc1_Average(ADC_Channel_1,100);
        temp=(float)adcx*(3.3/4096);
        Detected_power=(24.36-56.75*(temp)+14.22*(temp*temp)-
4.07*(temp*temp*temp))*(-10);
        printf("The Microwave Radiation Power= %f dBm\n",Detected_power/10*(-1));
        printf("The dc voltage= %f V\n",temp);
        if((int)Detected_power<=130)
        {
            if((int)Detected_power<0)Detected_power*=(-1);
            HMISends("page button0_2");
            HMISendb(0xff);
                HMI_SendNum(USART3,"button0_2.x0.val=-",18,(int)Detected_power,3);
            return;
        }
        else if((int)Detected_power<=300)
        {
            HMISends("page button0_1");
            HMISendb(0xff);
                HMI_SendNum(USART3,"button0_1.x0.val=-",18,(int)Detected_power,3);
            return;
        }
        HMI_SendNum(USART3,"button0.x0.val=-",16,(int)Detected_power,3);
        return;
    }
```

```
	else if(button==2)
	{
		adcx=Get_Adc1_Average(ADC_Channel_1,100);
		temp=(float)adcx*(3.3/4096);
		Detected_power=(24.36-56.75*(temp)+14.22*(temp*temp)-
4.07*(temp*temp*temp))*(-10);
		printf("The Microwave Radiation Power= %f dBm\n",Detected_power/10*(-1));
		printf("The dc voltage= %f V\n",temp);
		if((int)Detected_power>=325)
		{
			HMISends("page button0");
			HMISendb(0xff);
				HMI_SendNum(USART3,"button0.x0.val=-",16,(int)Detected_power,3);
			return;
		}
		else if((int)Detected_power<=130)
		{
			if((int)Detected_power<0)Detected_power*=(-1);
				HMISends("page button0_2");
			HMISendb(0xff);
			HMI_SendNum(USART3,"button0_2.x0.val=-",18,(int)Detected_power,3);
			return;
		}
	HMI_SendNum(USART3,"button0_1.x0.val=-",18,(int)Detected_power,3);
		return;
	}
	else if(button==3)
	{
		adcx=Get_Adc1_Average(ADC_Channel_1,100);
		temp=(float)adcx*(3.3/4096);
		Detected_power=(24.36-56.75*(temp)+14.22*(temp*temp)-
4.07*(temp*temp*temp))*(-10);
		printf("The Microwave Radiation Power= %f dBm\n",Detected_power/10*(-1));
		printf("The dc voltage= %f V\n",temp);
		if((int)Detected_power>=325)
		{
			HMISends("page button0");
			HMISendb(0xff);
			HMI_SendNum(USART3,"button0.x0.val=-",16,(int)Detected_power,3);
				return;
		}
		else if((int)Detected_power>=155)
		{
			HMISends("page button0_1");
```

```
			HMISendb(0xff);
					HMI_SendNum(USART3,"button0_1.x0.val=-
",18,(int)Detected_power,3);
			return;
		}
		if((int)Detected_power<0)Detected_power*=(-1);
				HMI_SendNum(USART3,"button0_2.x0.val=-",18,(int)Detected_power,3);
		return;
	}
}

void Bluebooth(void)
{
	if(button==7)
	{
		LED1=0;
		BLE=1;
		HMISends("button1.t0.txt=\"OPEN\"");
		HMISendb(0xff);
		return;
	}
	if(button==8)
	{
		LED1=1;
		BLE=0;
		HMISends("button1.t0.txt=\"CLOSE\"");
		HMISendb(0xff);
		return;
	}
}

void Batterydisplay(void)
{
	if(button==6)
	{
		adcx_2=Get_Adc2_Average(ADC_Channel_9,120);
		temp_2=(float)adcx_2*(3.3/4096);
		if(BLE==0)
		{
			Battery_rate_f=(1-(8.15-temp_2*2.55)/1.03)*100;
			if(percentage-Battery_rate_f>=1.8)
				percentage-=1;
			if(Battery_rate_f-percentage>=1.8)
				percentage+=1;
```

```
            Battery_rate=(int)percentage;
                    if(Battery_rate>100)
            Battery_rate=100;
        if(Battery_rate<=0)
        {
            HMISends("page button3_1");
            HMISendb(0xff);
            return;
        }
        HMI_SendNum(USART3,"button3.n0.val=",15,Battery_rate,3);

HMI_SendNum(USART3,"button3.n1.val=",15,BAT_Usagetime[Battery_rate]/60,2);

HMI_SendNum(USART3,"button3.n2.val=",15,BAT_Usagetime[Battery_rate]%60,2);
    }
    else
    {
        Battery_rate_f=(1-(8.15-(temp_2*2.55+0.045))/1.03)*100;
        if(percentage-Battery_rate_f>=1.8)
            percentage-=1;
        if(Battery_rate_f-percentage>=1.8)
            percentage+=1;
        Battery_rate=(int)percentage;
        if(Battery_rate>100)
            Battery_rate=100;
        if(Battery_rate<0)
            Battery_rate=0;
        HMI_SendNum(USART3,"button3.n0.val=",15,Battery_rate,3);

HMI_SendNum(USART3,"button3.n1.val=",15,(int)(BAT_Usagetime[Battery_rate]*0.85/60
),2);

HMI_SendNum(USART3,"button3.n2.val=",15,(int)(BAT_Usagetime[Battery_rate]*0.85)%
60,2);
    }
    return;
}
if(button==9)
{
    adcx_2=Get_Adc2_Average(ADC_Channel_9,120);
    temp_2=(float)adcx_2*(3.3/4096);
    if(BLE==0)
    {
        Battery_rate_f=(1-(8.15-temp_2*2.55)/1.03)*100;
```

```
			if(percentage-Battery_rate_f>=1.8)
				percentage-=1;
			if(Battery_rate_f-percentage>=1.8)
				percentage+=1;
				Battery_rate=(int)percentage;
			if(Battery_rate>0)
			{
					HMISends("page button3");
				HMISendb(0xff);
				HMI_SendNum(USART3,"button3.n0.val=",15,Battery_rate,3);

HMI_SendNum(USART3,"button3.n1.val=",15,BAT_Usagetime[Battery_rate]/60,2);

HMI_SendNum(USART3,"button3.n2.val=",15,BAT_Usagetime[Battery_rate]%60,2);
				return;
			}
		}
		else
		{
			Battery_rate_f=(1-(8.15-(temp_2*2.55+0.045))/1.03)*100;
			if(percentage-Battery_rate_f>=1.8)
				percentage-=1;
			if(Battery_rate_f-percentage>=1.8)
				percentage+=1;
				Battery_rate=(int)percentage;
			if(Battery_rate>0)
			{
				HMISends("page button3");
				HMISendb(0xff);
				HMI_SendNum(USART3,"button3.n0.val=",15,Battery_rate,3);

HMI_SendNum(USART3,"button3.n1.val=",15,(int)(BAT_Usagetime[Battery_rate]*0.85/60),2);

HMI_SendNum(USART3,"button3.n2.val=",15,(int)(BAT_Usagetime[Battery_rate]*0.85)%60,2);
					return;
			}
		}

	}
}
```
